# Fabricating CAD/CAM Implant-Retained Mandibular Bar Overdentures: A Clinical and Technical Overview

**DOI:** 10.1155/2017/9373818

**Published:** 2017-03-15

**Authors:** Chui Ling Goo, Keson Beng Choon Tan

**Affiliations:** ^1^Jabatan Pergigian Operatif, Fakulti Pergigian, University Kebangsaan Malaysia, Jalan Raja Muda Aziz, 50300 Kuala Lumpur, Malaysia; ^2^Faculty of Dentistry, National University of Singapore, 11 Lower Kent Ridge Road, Singapore 119083

## Abstract

This report describes the clinical and technical aspects in the oral rehabilitation of an edentulous patient with knife-edge ridge at the mandibular anterior edentulous region, using implant-retained overdentures. The application of computer-aided design and computer-aided manufacturing (CAD/CAM) in the fabrication of the overdenture framework simplifies the laboratory process of the implant prostheses. The Nobel Procera CAD/CAM System was utilised to produce a lightweight titanium overdenture bar with locator attachments. It is proposed that the digital workflow of CAD/CAM milled implant overdenture bar allows us to avoid numerous technical steps and possibility of casting errors involved in the conventional casting of such bars.

## 1. Introduction

Implant dentistry has given hope to edentulous patients in obtaining a prosthesis which is adequately retained, stable, and comfortable as well. McGill Consensus has suggested that the first line of treatment for an edentulous mandible is 2-implant-retained overdentures [1]. Elderly patients using mandibular implant overdentures report better oral-health related quality of life (OHQOL) and general health outcomes [2–4].

The 2 implants with either locators or connecting bar allow for an implant-retained, but mucosa-supported overdenture on the mandible. The bar allows for free rotation during dorsal loading with twist-free load transmission to the implants [5]. This design still depends largely on the mucosal support for its stability. However, some patients with knife-edge or sensitive mandibular ridges are better suited as candidates for a prosthesis which is completely supported by implants [6]. As such, in these patients, a fixed prosthesis is the preferred design choice. However, due to the severe loss of mandibular alveolar ridge, provision of the fixed prosthesis would be inadequate to compensate for the loss of both soft and hard tissue, thus compromising the facial aesthetics of the patient. In this case, implant-supported overdentures would satisfy both aesthetics and prosthesis retention needs.

Implant overdenture bars are traditionally fabricated using the lost-wax technique and conventional casting method, which is time-consuming and labour-intensive. Alternatively, the overdenture bar framework can be fabricated using the CAD/CAM method. Fabrication of cast one-piece implant frameworks are occasionally fraught with problems such as possible misfits and porosities [7]. There is some debate with regard to the clinical significance of passive fit and the acceptable range of the misfit values [8, 9]. However, some studies will concur that prosthetic complications such as screw loosening and abutment fractures may be related to poor framework fit [10, 11]. As such, it would be sensible when fabricating such frameworks, to minimise such inaccuracies. With cast implant frameworks, these problems could be rectified with certain additional laboratory steps such as sectioning and soldering or laser welding [12], but it would add on to the fabrication workflow, thus increasing time and costs. This case demonstrates the simplification of laboratory workflow by application of computer-aided design and computer-aided manufacturing (CAD/CAM) in the fabrication of the overdenture framework bar.

## 2. Case Presentation

### 2.1. History and Examination

A 62-year-old healthy male patient presented with complaints of loose and uncomfortable mandibular dentures. The dentures were relined twice in an attempt to relieve the symptoms but were unsuccessful. He requested for a more stable and comfortable replacement.

**Figure 1:**
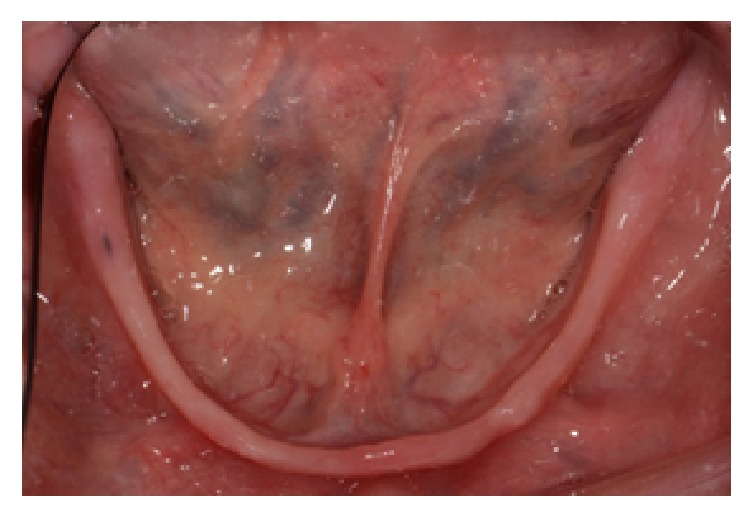
Edentulous mandibular arch with knife-edge ridge.

Clinical and radiographic examinations revealed a Class III skeletal pattern with edentulous maxillary and mandibular ridges. The maxillary arch had U-shaped rounded ridges. The mandibular arch was also U-shaped with large curvature and knife-edge ridges (Figure 1). According to the American College of Prosthodontists Prosthodontic Diagnostic Index (ACP PDI) for classification of complete edentulism, the patient was categorized as Class IV, due to the Class III maxillomandibular relationship and need for preprosthetic surgery to allow for adequate prosthodontic function [13].

**Figure 2:**
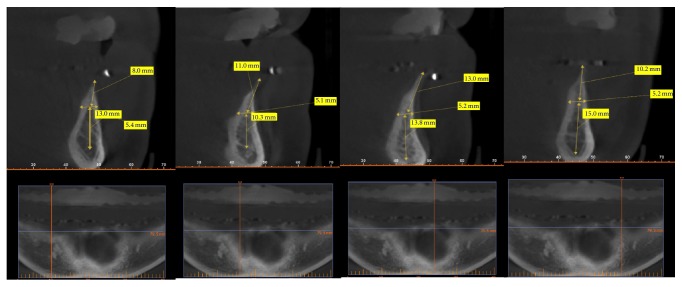
Cone beam computed tomography (CBCT) findings showing very thin coronal portion of the alveolar ridge.

His existing prostheses were fabricated a year ago with both anterior and posterior crossbite teeth setup. The retention and stability of the maxillary denture were good while the mandibular denture lacked stability in function. The CBCT scan revealed a very thin Class IV type of alveolar ridge (knife-edge) (Figure 2). A set of interim dentures were processed using thirty-degree denture teeth (Dentacryl HXL, Dentsply USA) and heat-polymerized acrylic resin (Lucitone 199, Dentsply USA) to test out the new diagnostic teeth setup in which the occlusal scheme prescribed for the patient was balanced articulation in posterior crossbite setup [14]. Based on this new diagnostic teeth setup, patient was deemed to have sufficient restorative space and patient accepted the correction of anterior crossbite.

### 2.2. Treatment

The clinical and radiographic findings were discussed with the patient and the treatment plan below was formulated with patient's agreement.

*Surgical Phase*

- Alveoloplasty to eliminate the knife-edge coronal portion of the anterior mandibular ridge
- Placement of four implants at the interforamina area of the mandible

*Prosthodontic Phase*

- CAD/CAM bar-supported, locator-retained complete overdenture for mandibular arch
- Conventional complete denture for maxillary arch

#### 2.2.1. Surgical Phase

**Figure 3:**
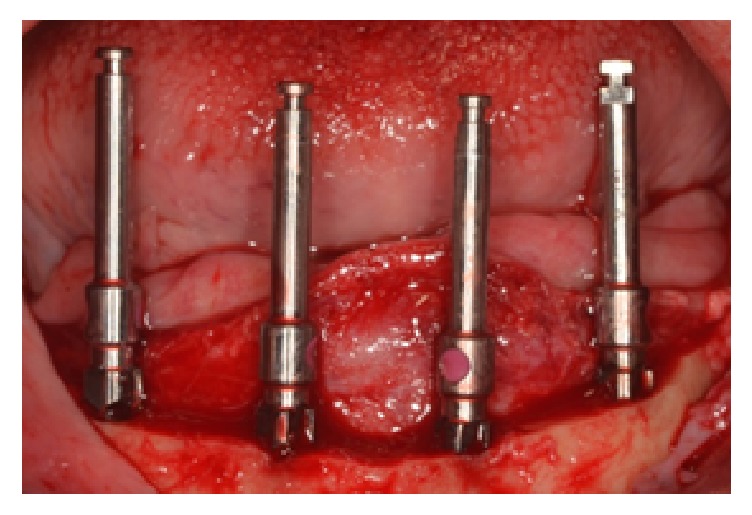
Alignment of the four implants' position.

**Figure 4:**
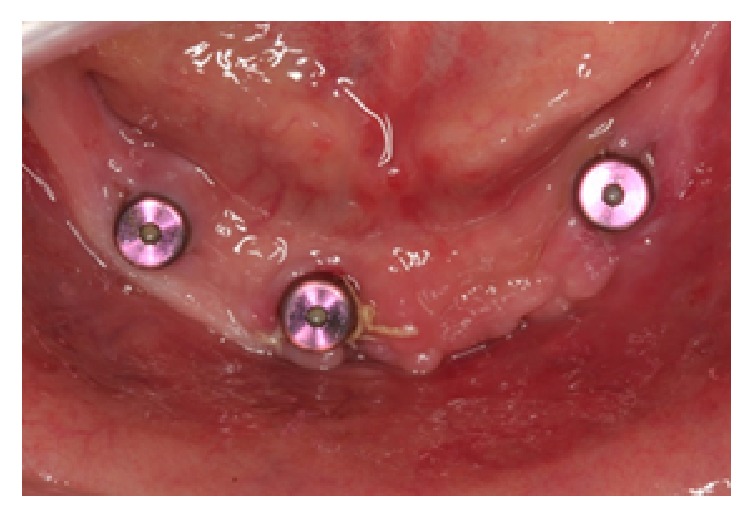
Three 5 mm healing abutments placed on the remaining implants.

**Figure 5:**
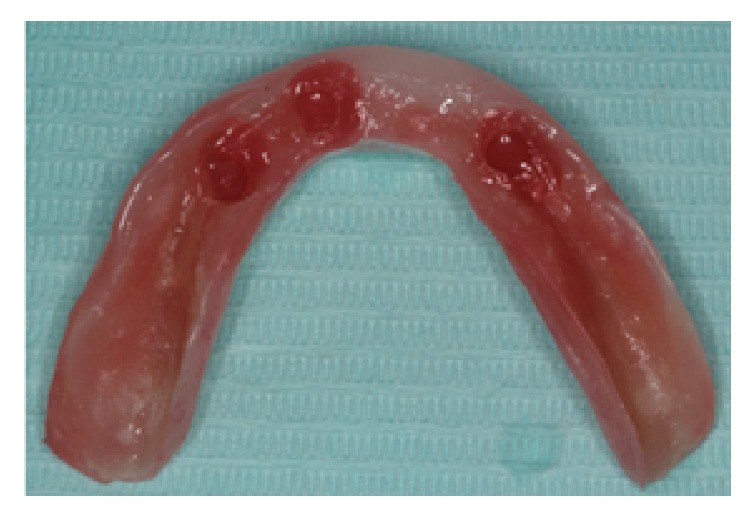
The intaglio surface of the mandibular interim denture is relined.

The proposed alveoloplasty was simulated on the working casts, with removal of approximately 8–10 mm of vertical bone height. The implant positioning was marked and drilled on the clear acrylic resin (Caulk Orthodontic Resin, Dentsply, USA) surgical guide. Based on the amount of alveolar ridge bone available, curvature of the arch, and teeth setup, four Nobel Replace Conical Connection NP 3.5 mm × 13 mm implants (Nobel Biocare, USA) were planned at the interforaminal region of the mandibular anterior region (Figure 3). Cover screws were placed on the implants and minor bone grafting using demineralised bovine bone matrix (BioOss, Geistlich, Switzerland) and barrier membrane (BioGide, Geistlich, Switzerland) was done to cover exposed threads at the #42 implant area. The interim dentures were relieved completely on the intaglio surface surgical area and relined with a soft tissue conditioning material (Visco-Gel, Dentsply USA). Patient was advised to omit the wear of mandibular dentures as much as possible. After 4 months, the second stage of surgery was done. During this appointment, the failure of #32 implant osseointegration was noted and this was confirmed by the radiolucency noted surrounding the implant. Subsequently, the failed implant was removed while 5 mm healing abutments replaced the cover screws of the other three implants (Figure 4). The soft lining material was removed from the intaglio surface and relined with a denture repair acrylic resin material (Rebase II, Tokuyama, Japan) (Figure 5).

#### 2.2.2. Prosthodontic Phase

Master impression of the maxilla and mandible was registered in Zinc Oxide Eugenol (SS White, USA) and polyether impression material (Impregum F, 3M ESPE, USA), respectively. The mandible arch impression utilised an open-tray technique with splinted impression copings [15]. After the impression copings were screwed onto the implants and verified with radiographs for complete seating, the copings were joined together by lacing the floss across the copings in a figure of “8” and subsequently strengthened with autopolymerization resin (GC Pattern Resin, Japan). After waiting for approximately 20 minutes (to allow for polymerization shrinkage), the impression was registered in polyether material.

From the master cast, a verification index was made by connecting the three impression copings with autopolymerizing resin (GC Pattern Resin, USA) and subsequently sectioned vertically for reassembly in the mouth [16]. The reassembled sections were then joined together with pattern resin. These reassembled impression copings were verified with the master cast. If there were any discrepancies with the master cast, the master impression should be repeated. In this patient, the verification jig confirmed the correct position of implants. The maxilla-mandibular relationship (MMR) was recorded with bite registration material (Regisil 2X, Dentsply USA), with the mandibular denture base sitting on the 5 mm healing abutments during this visit.

**Figure 6:**
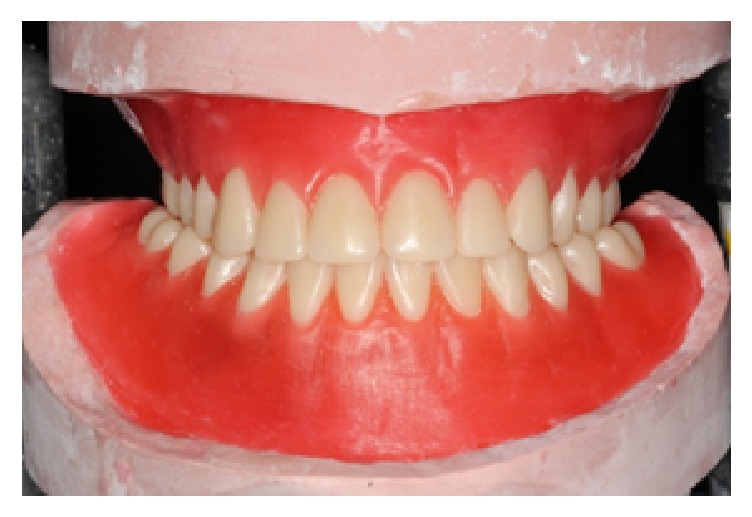
The teeth setup in wax.

**Figure 7:**
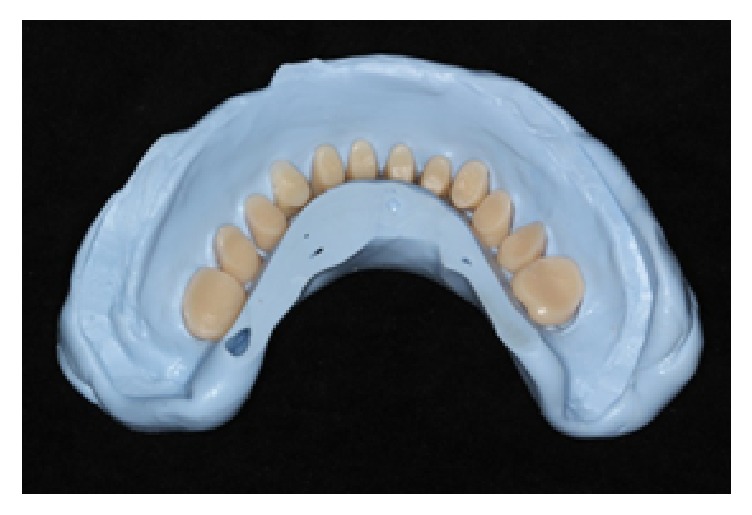
Silicone index registering the occlusal position of the denture teeth.

**Figure 8:**
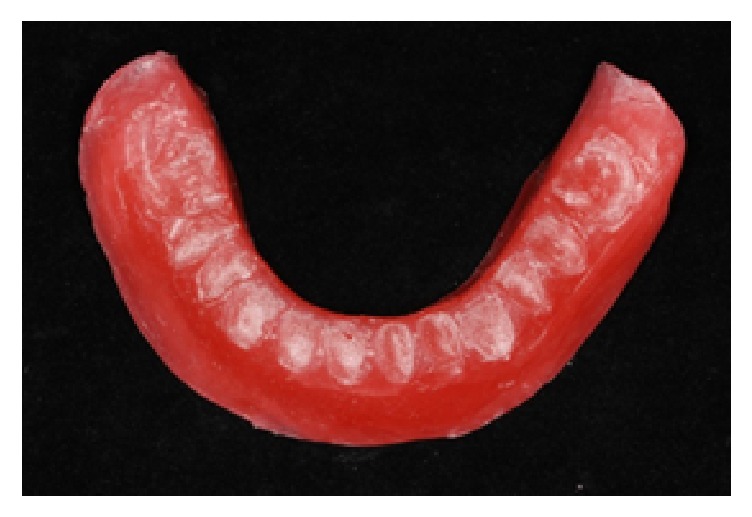
Wax denture base with the denture teeth removed.

**Figure 9:**
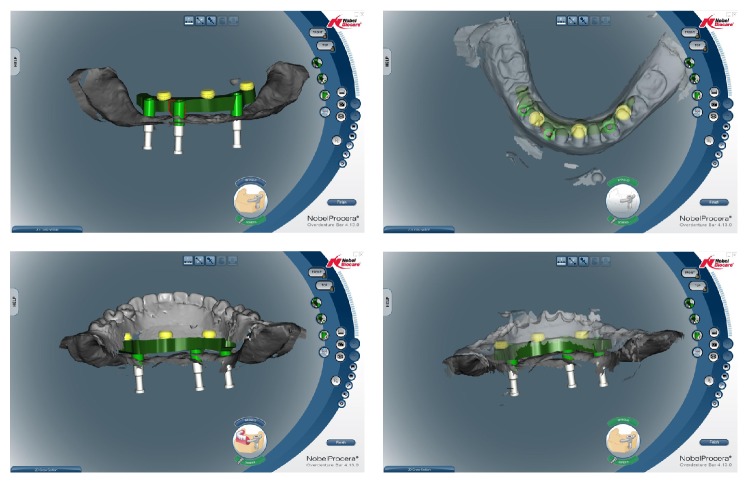
Design of the framework using the Nobel Procera software.

The master casts were then mounted according to the MMR on a semiadjustable articulator (Dentatus Articulator ARH, Sweden) and the teeth setup was done (Figure 6). Another appointment for clinical try-in was done to check the aesthetics and occlusion for the patient. After the patient accepted the clinical try-in, the dentures were then brought back to the laboratory to have an occlusal silicone index fabricated to locate the position of the mandibular denture teeth (Figure 7). Three items were required for the Nobel Procera scan: (1) master cast model, (2) wax denture base with teeth setup, and (3) wax denture base without teeth setup (the intaglio surface of teeth) (Figure 8). The CAD system is then able to superimpose the images or remove the superimposition at will during the designing process. Using the denture base without teeth setup, the actual amount of space available for the bar framework can be visualised so that the space for denture teeth is undisturbed. The amount of taper can be fixed to avoid any undercuts for the bar. Attachments can also be added during this stage. The planned implant suprastructure can be checked if it fits within the restricted space by superimposing the images of the dentures, with teeth or without teeth. Similarly, the thickness of the denture base can also be checked to be sufficient for strength. Once the design is confirmed, the design is directly sent to the Nobel Procera Production Facility in Japan through the software itself. For this case, the process of scanning and designing the suprastructure was done using the Nobel Procera scanner and software (Figure 9) located at the dental centre's in-house laboratory; thus the design could be controlled by the clinician. Alternatively, if the software was not available, the scanning and design could be carried out in the milling centre and the design has to be approved by the clinician before milling. The lead-time for the fabrication of the framework was 10–14 working days.

**Figure 10:**
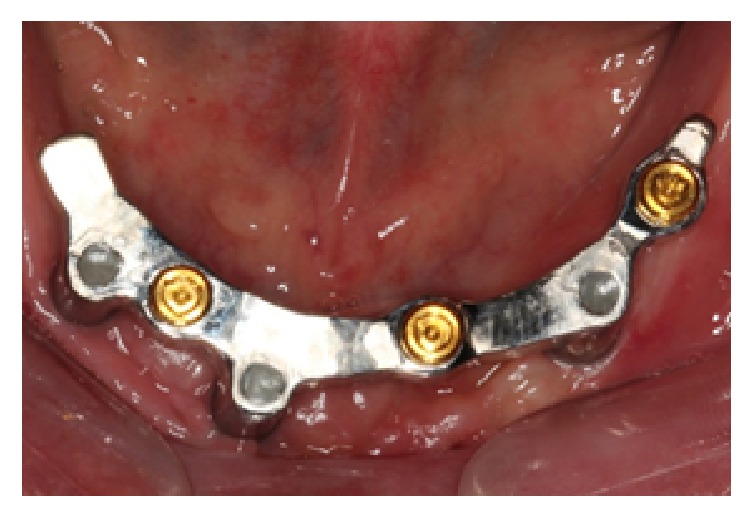
Milled implant framework tried in the mouth.

**Figure 11:**
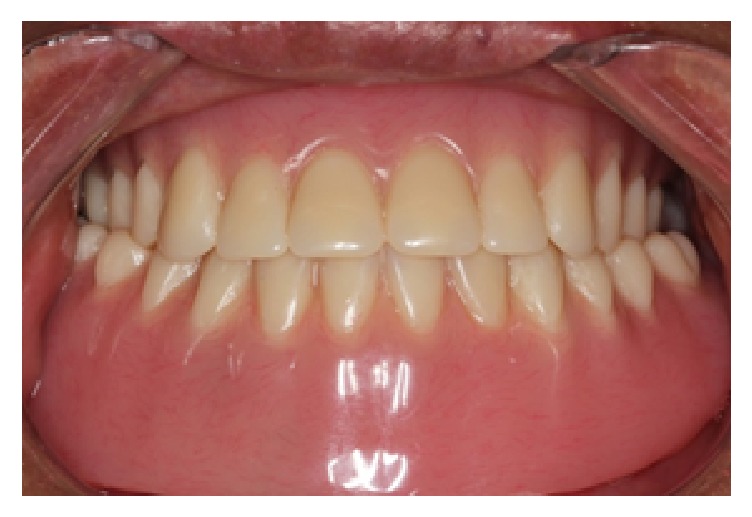
Final prosthesis in the mouth.

The implant bar framework was then received and tried in the patient's mouth (Figure 10). The fit of the framework was verified using the single screw test (Sheffield test) [17]. For nonvisible implant abutment junctions, a periapical radiograph was taken to verify the fit [18]. Once the fit of framework was confirmed, the milled bar was brought back into the laboratory for the denture processing stage. The milled bar was first secured to the master cast. White blockout rings were placed and the metal housing processing male caps were snapped onto each female part. The undercuts below the bar were blocked out using silicone. The occlusal index with teeth was placed back onto the model and wax was flowed into the pink acrylic denture space. The maxillary and mandibular dentures were then processed as per normal in heat-polymerized acrylic resin. After the dentures were deflasked, the male processing caps were removed and replaced with a light retention replacement male cap (blue) (Zest Anchor, USA). The dentures were issued to the patient with accompanying home care instructions (Figure 11). After 2 weeks, the patient was reviewed and he reported overall satisfaction with the prosthesis.

## 3. Discussion

A biomechanically sound treatment plan to restore an edentulous mandible with implants includes placing implants at key implant positions and increasing the number of implants to increase surface area for stress distribution [19]. For practical reasons, the number of implants needed to restore an edentulous mandible also depends on patient's expectations (in terms of retention and stability), financial ability, and desired outcome. For this patient, his initial wish was to have fixed implant prosthesis as costs were not a major issue for him. However, due to the lack of bony support available for advocating fixed implant prostheses in both maxilla and mandibular arches, a compromised plan was proposed, after ruling out more extensive treatment to achieve the fixed implant prosthesis plan.

A large curvature on a U-shaped mandible allows for adequate placement of four implants and connecting bar. A rigid bar connecting multiple implants and a cast metal framework would reinforce the biomechanical strength of the foundation and ensures stability of the denture base [20, 21]. The design for implant overdentures depends on preferred method of attachment, availability of interocclusal space, and amount of support required from the implant and ridge mucosa.

The loss of the #32 implant prior to the loading of implants was due to failure to establish osseointegration. Primary stability was noted for all implants during the surgery and patient was covered with antibiotics postoperatively. There were no signs and symptoms of infection during the healing period but the asymptomatic failed implant was noted to be mobile at second stage surgery. A review by Esposito et al. [22] stated possible aetiologic factors for early failure of implants including infection, impaired healing, and disruption of a weak-bone implant interface. The possible reason in this patient was impaired healing due to overheating of bone. The dense homogenous compact basal bone at the anterior region could have sustained damage during the alveoloplasty procedure, causing localised necrosis of the bone.

The subsequent treatment plan was discussed with patient, either to redo #32 implant and continue with initial plan or to proceed with the prosthetic phase with only three implants. Unfortunately, patient refused replacement of the failed implant as patient was not keen to go through another round of surgery and extended waiting duration. The initial prosthetic plan was to include four implants splinted together with a milled bar to distribute the loading stresses. Two implants were deemed insufficient, as the implants were narrow diameter implants. A bar placed anterior to the interconnecting line between the two implants could cause extremely large compressive and tensile stress concentrations in the bone around the implants. Therefore, in such situations, it was not advisable to connect the implants. If a bar-clip attachment was to be used, additional implants were required to be placed in the frontal region [23]. Thus, for this patient, a compromised treatment plan was agreed upon to utilise the remaining three implants for the mandibular bar. The bar was indicated to resist lateral load by providing cross arch stabilization. The bar also helped to improve the stability of the prosthesis by providing a distal cantilever in which the attachment of the overdenture can be placed posterior to the distalmost implants. The minimum space for a bar-supported overdentures is 13-14 mm, while for locator-retained overdentures the minimum interocclusal space was between 8 and 10 mm. In this case, there was adequate space required for the height of the milled bar, locator attachments, and denture base as alveoloplasty had been performed. While locator attachments are primarily designed to withstand lateral loading and distribute the stresses, in this case of milled bar and locator attachments, the locators no longer perform that function. Instead, the locators are used for retention of overdentures to the milled bars and to provide some vertical resiliency during occlusion. Support for the mandibular overdenture was gained through the close adaptation of the denture base to the milled bar and in the posterior region, minimally mucosal-supported.

The position of the locators on the milled bar is slightly lingual and does not follow the arch curvature exactly as the patient had skeletal Class III pattern with broad mandibular arch, while our teeth setup attempts to mask this discrepancy by converting the anterior occlusion into a Class I occlusion. The ability to superimpose multiple views on the CAD software allows us to visualise a suitable location for the locators and this is certainly an advantage when compared to the conventional use of sectioned matrices on the working model.

Another benefit of using the milled bar with locator attachments is that the overdenture platform is raised to ease the insertion and removal of the prosthesis with less interference from the surrounding soft tissue, for example, lips or buccal mucosa, flopping over the attachments.

The advantages of CAD/CAM implant frameworks versus one-piece cast frameworks are manifold. The accuracy and fit of these CAD/CAM frameworks have been shown to be more accurate than one-piece cast framework by a number of studies [24–27]. The costs of CAD/CAM implant frameworks are potentially lower than one-piece cast frameworks as titanium alloy rather than noble alloy is used for the CAD/CAM framework. Titanium alloy frameworks are also lighter in weight than the noble alloy frameworks for the same design due to the density of the metal itself. Moreover, CAD/CAM frameworks do not need a separate order of abutments as the abutments are milled as part of the CAD/CAM framework. The locator female attachments are also screwed into a milled screw base on the bar, with perfect fit. Thus, all the locators will have the same insertion axis as the milled bar and, in the process, reduce the wear of the locator replacements. In the conventional method of casting the framework, a milling bur drills a hole through the bar resin pattern. A locator paralleling plastic post is then mounted onto the milling machine handpiece to ensure parallel placement of the attachment and the locators secured with autopolymerizing acrylic resin. This process has room for error when it is carried out by inexperienced technicians; thus the CAD/CAM milled framework holds the upper hand in accuracy of the locator placement. In the event where the need for redo of the implant framework is required, the same design file can be utilised to fabricate a new framework without remaking the impression.

While the CAD/CAM approach presents a viable alternative to the conventional processing of splinted implant framework, the costs are at the higher end of the scale at this point of time and prohibitive for certain groups of patients. In these patients, prescribing cost effective implant-supported prosthesis would probably be the unsplinted implant-retained prosthesis, for example, using ball and socket attachments or locator attachments. However, data comparing splinted versus unsplinted implant prosthesis found that the unsplinted prosthesis designs need more prosthetic maintenance [28]. As such, this information should be conveyed to the patient while discussing the pros and cons of each treatment option.

## 4. Conclusion

The restorative work process for implant-supported prostheses to rehabilitate an edentulous mandible has been simplified with the use of CAD/CAM technology. Commercialization of the CAD/CAM technology and increase in supply and demand forces will eventually lower the costs involved. In the future, it is foreseeable that CAD/CAM technology will further enhance the delivery of dental services to the aging population with edentulism.
